# OVCCA Web Application as Supplementary Material to Facilitate Health Literacy Regarding Carcinogenic Human Liver Fluke: A Randomized Controlled Trial in Thailand

**DOI:** 10.31557/APJCP.2021.22.9.3045

**Published:** 2021-09

**Authors:** Oranard Wattanawong, Tiwakron Prachaiboon, Thirayu Meererksom, Nathkapach Kaewpitoon Rattanapitoon, Schawanya Kaewpitoon Rattanapitoon, Pannee Banchonhattakit, Sarawut Boonsuk, Thongroo Kophachon

**Affiliations:** 1 *Division of General Communicable Diseases, Department of Disease Control, Ministry of Public, Health, Nonthaburi, Thailand. *; 2 *Department of Public Health, Faculty of Public Health, Khon Kaen University, Khon Kaen, Thailand. *; 3 *Parasitic Disease Research Center, Suranaree University of Technology, Nakhon Ratchasima, Thailand. *; 4 *Faculty of Public Health, Valaya Alongkorn Rajabhat University, Thailand. *; 5 *Department of Health, Ministry of Public Health, Khon Kaen, Thailand. *

**Keywords:** Health literacy, Carcinogenic human liver fluke, Opisthorchis viverrini, OVCCA web application, Thailand

## Abstract

Liver fluke, *Opisthorchis viverrini*, is associated to cholangiocarcinoma which is found frequently in some areas of Southeast Asian countries particularly in Thailand, Lao People Republic Democratic, Cambodia. This study sought to investigate the effects of an *O. viverrini *and cholangiocarcinoma (OVCCA) web application to facilitate health literacy regarding *O. viverrini *in Northeast Thailand. Methods: A randomized controlled trial study was performed among an intervention group (n=63) and a control group (n=63) during a one-year period from July 2019 to May 2020. The intervention group received the health literacy promotion program of *O. viverrini *information through the OVCCA web application for 6 weeks. The control group received an activity package from the public health department. The success of the program was evaluated at week 24 after the groups finished the last activity. ANCOVA, t-test and multiple logistic regression were used for data analysis for both groups. Results: The scores for knowledge; ability to access, understand, appraise, and apply information; motivation for protection; and practice of *O. viverrini *prevention were significantly higher for the intervention group than for the control group. The results indicated that a health literacy promotion program through an OVCCA web application could be advantageous for preventing and controlling *O. viverrini *infection. Conclusion: This intervention may be used as a potential strategy and guideline for self-care and health promotion in other endemic areas.

## Introduction

Infection caused by the liver fluke, *Opisthorchis viverrini*, is a neglected tropical disease that affects health and poverty, particularly in Southeast Asian countries such as Thailand, Lao People’s Democratic Republic, Cambodia, and the central part of Vietnam (Hotez et al., 2015). Nine million people per year suffer from *O. viverrini *infection (Sithithaworn et al., 2014). *O. viverrini *infection is associated with hepatobiliary diseases, including cholangiocarcinoma, mainly in northeastern Thailand and Laos because people in those areas extensively consume raw or half-cooked fish (Kaewpitoon et al., 2008; Chudthaisong et al., 2015; Saiyachak et al., 2016). In Thailand, more than 6 million people are at risk of liver fluke infection (Sithithaworn et al., 2012), which has been found to have the highest prevalence in the northeast (16.6%) and north (10%) (Wongsaroj et al., 2014). The infection is frequently found in men over 35 years of age and in agricultural occupations (Kaewpitoon et al., 2015). The infection results from the consumption of raw or undercooked freshwater cyprinoid fish. Many people dose with praziquantel as a treatment for an episode of the infection, and they still have a high risk for them of long-term consequences (Sithithaworn et al., 2012; Saengsawang et al., 2013). Effective prevention and control campaigns are needed to decrease the problem in the population at risk of disease. Health literacy (HL) represents the cognitive skills and social skills that determine the motivation and ability of individuals to promote and maintain good health (World Health Organization, 1998). People’s competencies to access, understand, judge and apply health information in healthcare, disease prevention, and health promotion are based on their HL level and the factors associated with the specific population (Denuwara and Gunawardena, 2017). HL is a concept used in healthcare, disease prevention, and health promotion settings. It includes the following concepts: (1) access refers to the ability to seek, find and obtain health information; (2) understand refers to the ability to comprehend the health information that is accessed; (3) appraise describes the ability to interpret, filter, judge and evaluate the health information that is accessed; and (4) apply refers to the ability to communicate and use the information to make a decision to maintain and improve health (Sørensen et al., 2012). Therefore, HL is a good way to combat serious liver fluke infections in epidemic areas. Ferrucci et al., (2021) suggest that this approach provides a basis for future artificial intelligence-based approaches. We examined HL for *O. viverrini *and found that the population at risk of infection from this liver fluke had a high level of understanding and good practices after intervention (Prachaiboon et al., 2021). Recently, mobile and web applications for health care have increased worldwide, including in Thailand. The sustainability of such an application depends on the use and function of mobile and web applications (Akhbardeh et al., 2015; Anderson et al., 2016; Radovic et al., 2016). A mobile application screening test has been used to screen for *O. viverrini *infection, and this tool proved to be simple, rapid and low cost (Kaewpitoon N et al., 2018). Therefore, this study aimed to investigate the effects of health literacy promotion programs and an OVCCA web application on the prevention of *O. viverrini *infection in Northeast Thailand.

## Materials and Methods

*Study design*

**Figure 1:**
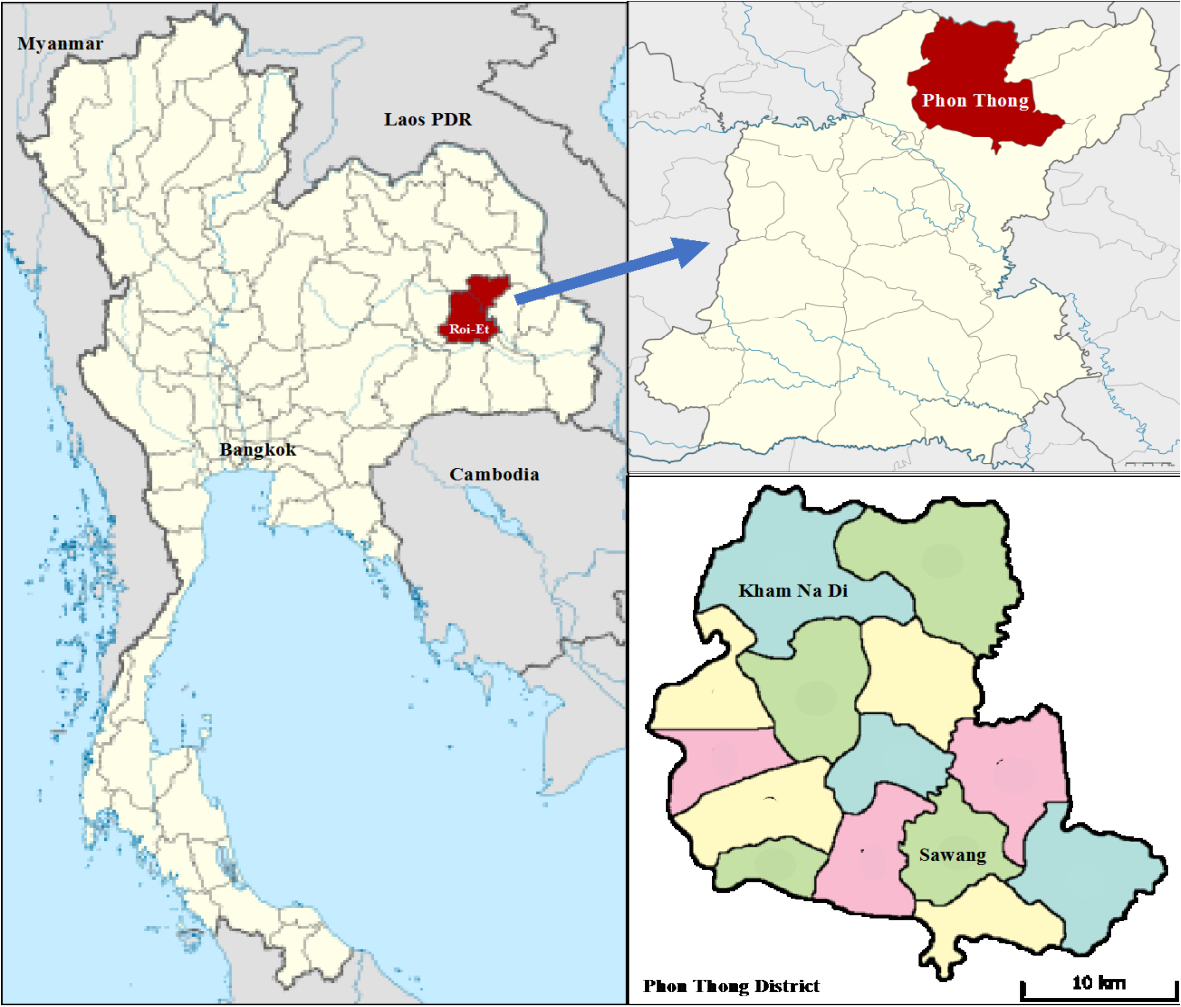
The Study Area, including the Kham Na Di and Sawang Subdistricts, where the Populations were Randomized into Intervention and Control Groups

**Figure 2:**
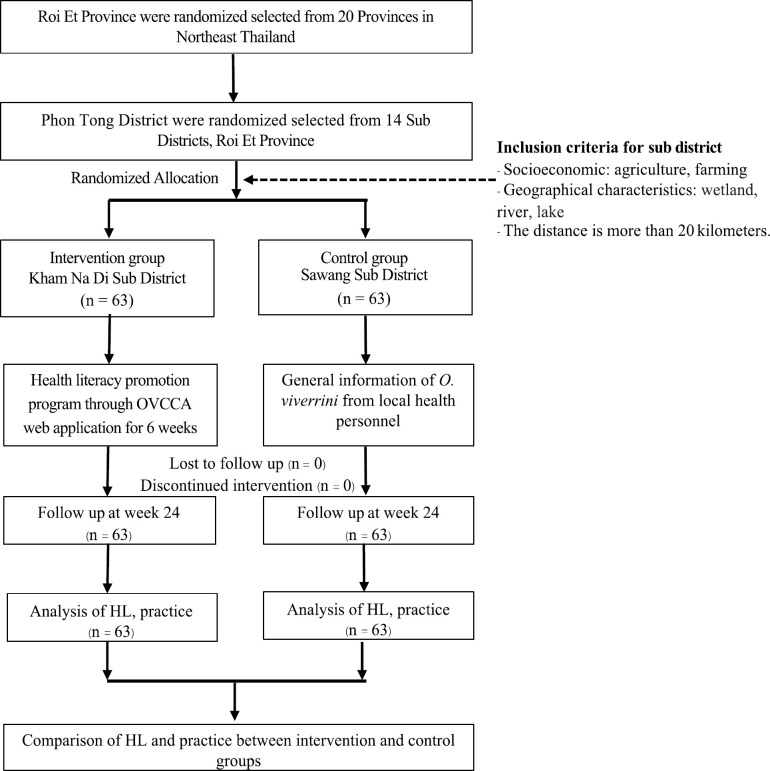
CONSORT Diagram of This Study

This randomized controlled trial was conducted in Roi Et Province, Northeast Thailand, for one year between July 2019 and May 2020 (Figure 1). A total population of 126 individuals living in Phon Tong District, with those in Kham Na Di Subdistrict as the intervention group and those in Sawang Subdistrict as the control group. The participants were selected through randomized allocation and completed self-administered questionnaires (Figure 2).

*Health literacy promotion program through OVCCA web application*

A health literacy promotion program (HLPP) and *O. viverrini* and cholangiocarcinoma (OVCCA) web application on *O. viverrini *prevention were performed among the intervention and control groups in Northeast Thailand. The intervention group received the HLPP of *O. viverrini *information through the OVCCA web application, while the control group received general information on *O. viverrini *from local health personnel as an annual campaign. The intervention group joined the 6-week HLPP through the OVCCA web application, which was developed as an open-source web application by the Department of Disease Control, Ministry of Public Health, which is affiliated with Parasitic Disease Research Center, Nakhon Ratchasima, Thailand. The application could be accessed by smartphone, tablet or computer. The HLPP program included 14 activities: week 1: (A1) Community participatory action with relationship building and (A2) Collecting data before intervention; self-checking using screening test, learning *O. viverrini *knowledge by (A3) animation of life cycle, signs and symptoms, prevention and control; (A4) VDO clip of pathogenesis and pathology, signs and symptoms, diagnosis, treatment, prevention and control; (A5) infographic images of morphology, life cycle, pathogenesis and pathology, diagnosis, prevention and control; (A6) pictures of morphology, life cycle, and diagnosis; (A7) health behavior daily record; week 2: A2-A7; (A8) lecture clip of *O. viverrini *knowledge; (A9) case-based learning using a VDO clip story of *O. viverrini *patients; week 3: A2-9; (A10) sharing experiences by role model by people who have succeeded in liver fluke prevention; week 4: A2-7; (A11) safety cooking demonstration using a VDO clip; week 5: A2-11; (A12) local folk songs (Esan song) clip related to liver fluke prevention; week 6: A2-12; (A13) home visit intervention group by health village volunteers, local health officers, and researchers; and (A14) meeting, reflecting, and returning data to communities and individuals so the supplemented intervention group could continue to receive health education.

*Assessment of health literacy promotion program through OVCCA web application*

A comprehensive health literacy questionnaire from the European Health Literacy Survey (HLS-EU-Q47) was applied and assessed all participants at week 24. This questionnaire was grounded in a conceptual framework and operationalized through a matrix with 7 dimensions, including four information-processing domains (finding, understanding, judging, and applying) and three health domains (health care, disease prevention, and health promotion) (Kaewpitoon et al., 2018). It consisted of 4 parts: 1) demographic characteristics; 2) knowledge about *O. viverrini*; 3) 4 HL skills: access information, understand information, appraise information, and apply information; and 4) practice of *O. viverrini *prevention and control. The questionnaire was validated by 7 experts and tested for reliability. The Kuder-Richardson 20 coefficient of knowledge about *O. viverrini *was 0.74. The Cronbach’s alpha coefficients of HL and *O. viverrini *infection practices were 0.87 and 0.76, respectively. Knowledge about *O. viverrini *infection was calculated and analyzed at three levels. The section on knowledge about *O. viverrini *consisted of 10 questions, with a correct answer = 1 and an incorrect answer = 0, and the scores were interpreted as high at 80% (8-10 points), moderate at 60%-79% (6-8 points), and low at 0-59% (0-5 points). The HL section included 47 questions that were interpreted as 0-25 points for inadequate, >25 to 33 points for problematic, >33 to 42 points for sufficient, and >42 to 50 points for excellent. The section on *O. viverrini *prevention and control practice had 10 questions that were interpreted as practice at 100% (10 points) and nonpractice at less than 100% (0-9 points). In this study, raw cyprinoid fish consumption was dichotomized (yes/no).

*Statistical analysis*

All analyses were performed using Stata version 10.0 (Stata Corp., College Station, TX). ANCOVA, t-test and multiple logistic regression were used to analyze raw cyprinoid fish consumption and HL scores by crude adjusted mean difference, adjusted relative ratios (ORadj) and 95% confidence interval (CI). All test statistics were two-sided, and a p-value of less than 0.05 was deemed statistically significant.

*Ethical Considerations*

This study was approved by the Ethics Committee in Human Research of Nakhon Ratchasima Health Provincial Office, Thailand 2018 (Reference no. NRPH041).

## Results

The majority of the intervention group were female (66.6%), aged 26-35 years (49.2%), with a high school education (54.0%), working in agriculture (famer) (52.4%), and with an income of 36-160 US dollar (42.9%). The majority of the control group were female (69.8%), aged 26-35 years (47.6%), with a high school education (57.1%), working in agriculture (famer) (49.2%), and with an income of 36-160 US dollar (41.3%) (Table 2). Thus, there were no differences in demographic data between the intervention and control groups. The results reveal that after the 6-week intervention and then at the 24-week follow-up, the mean scores of knowledge (p-value=0.001), ability to access information (p-value = 0.018), ability to understand information (p-value = 0.002), ability to appraise information (p-value = 0.038), ability to apply information (p-value = 0.043), and motivation for protection (p-value = 0.005) of *O. viverrini *prevention and control were higher for the intervention group than for the control group, and the difference was statistically significant (Table 1). In addition, the adjusted odds ratios (ORadj) of raw fish consumption for the health literacy promotion program and OVCCA web application for the prevention of *O. viverrini *infection were analyzed for both the intervention group and the control group, and the results indicated that after the intervention, the mean scores of practice for *O. viverrini *prevention were higher for the intervention group than for the control group (ORadj = 2.91: 95% CI: 1.11-7.67; p-value = 0.030), and the difference was statistically significant (Table 3).

**Table 1:** Background Variables in Intervention and Control Groups

Variables	Intervention group n (%)	Control group n (%)
Gender		
Male	21 (33.4)	19 (30.2)
Female	42 (66.6)	44 (69.8)
Age group (years)		
18-25	2 (3.2)	3 (4.8)
26-35	31 (49.2)	30 (47.6)
36-45	15 (23.8)	16 (25.4)
46-55	10 (15.8)	7 (11.1)
56-65	2 (3.2)	5 (7.9)
>65	3 (4.8)	2 (3.2)
Education		
Illiterate Primary	21 (33.3)	17 (27.0)
Secondary	34 (54.0)	36 (57.1)
Academic	8 (12.7)	10 (15.9)
Job status		
Housewife	12 (19.0)	14 (22.2)
Employed	6 (9.5)	5 (7.9)
Famer	33 (52.4)	31 (49.2)
Government officer	5 (7.9)	8 (12.7)
Trade	7 (11.0)	5 (7.9)
Family income ($, per month)		
<35	7 (11.1)	6 (9.5)
36–160	27 (42.9)	26 (41.3)
161–350	15 (23.8)	17 (27.0)
351–500	9 (14.3)	7 (11.1)
501–650	5 (7.9)	7 (11.1)
Raw or undercooked fish consumption		
Yes	58 (92.1)	57 (90.5)
No	5 (7.9)	6 (9.5)

Data presented as frequency (%).

**Table 2:** Results of Mean ± Standard Deviation and Adjusted Mean Difference, with 95% Confidence Intervals Between Intervention Group and Control Group among Health

Variables	Duration	Intervention group		Control group		Adj. mean difference	95% CI	P-value
		Mean	SD	Mean	SD			
Knowledge	Baseline	8.3	0.28	8.6	0.14			
	Follow	9.1	0.16	8.6	0.14	0.64	0.26 - 1.02	0.001
Ability to access information	Baseline	18.5	1.1	17.3	1.38			
	Follow	22.7	1.25	17.9	1.4	4.55	0.80 - 8.30	0.018
Ability to understand information	Baseline	26.4	1.12	26.8	1.23			
	Follow	29.8	1.23	25.1	1.25	5.49	2.11 - 8.87	0.002
Ability to appraise information	Baseline	24.4	0.84	25.7	0.97			
	Follow	28.9	1.29	26.6	0.96	3.01	0.16 - 5.86	0.038
Ability to apply information	Baseline	30.6	1.28	30.2	1.52			
	Follow	34.6	1.42	32.1	1.43	2.26	0.51 - 6.04	0.043
Protection motivation theory	Baseline	73.6	1.24	71.6	1.24			
	Follow	77.3	1.47	71.3	1.23	4.89	1.52 - 8.26	0.005

**Table 3:** Odds Ratios (ORadj) of Raw Fish Consumption and 95% Confidence Intervals for Health Literacy Promotion Program and OVCCA Web Application for the Prevention of *O. viverrini* Infection Between the Intervention Group and Control Group Using Multiple Logistic Regression

Prevention of O. viverrini infection	Intervention group		Control group		Crude OR	Adj. OR	95% CI	P-value
	n	%	n	%				
Baseline								
nonpractice	58	92.1	57	90.5	1	1	1	
practice	5	7.9	6	9.5	0.81	0.89	0.23-3.33	0.864
Follow								
nonpractice	45	71.4	55	87.3	1	1	1	
practice	18	28.6	8	12.7	2.75	2.91	1.11-7.67	0.03

**Figure 3:**
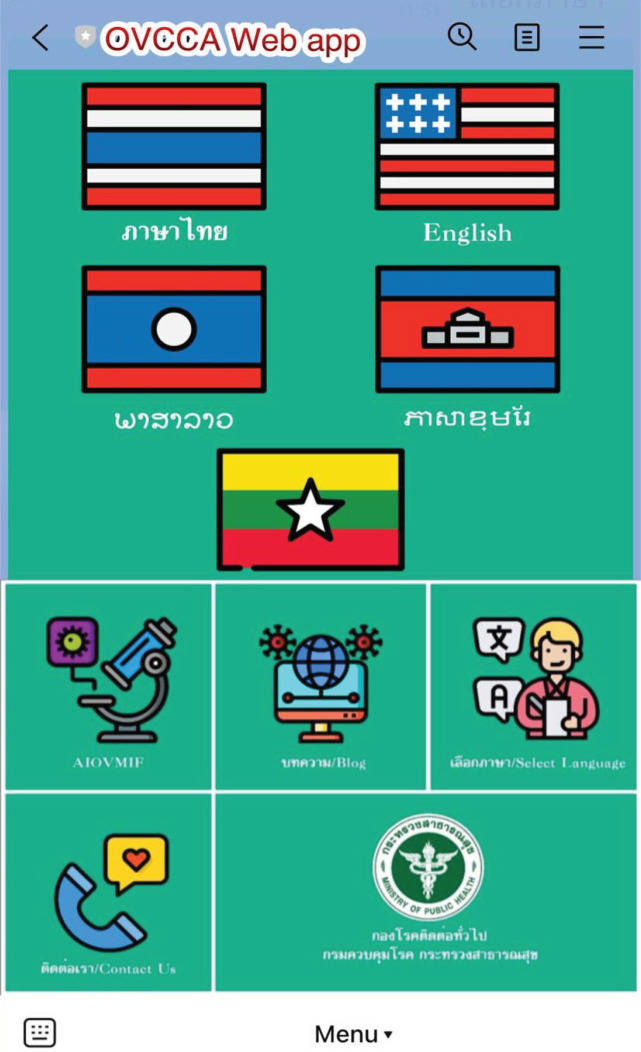
OVCCA Web Application; an Open-Source Web Application was Developed by the Department of Disease Control, Ministry of Public Health, which is Affiliated with the Parasitic Disease Research Center, Nakhon Ratchasima, Thailand

## Discussion

Infection by the liver fluke, *O. viverrini*, is associated with hepatobiliary diseases, including cholangiocarcinoma (Kaewpitoon et al., 2008; Chudthaisong et al., 2015; Saiyachak et al., 2016), and more than 9 million people per year suffer *O. viverrini *infection in Thailand (Sithithaworn et al., 2012). The infection results from the consumption of raw or undercooked freshwater cyprinoid fish, and many people suffer long-term consequences (Saengsawang et al., 2013). Prevention and control campaigns are needed to decrease the problem. HL represents cognitive skills and social skills related to people’s competency to access, understand, judge and apply health information in healthcare, disease prevention, and health promotion (Denuwara and Gunawardena, 2017; Duong et al., 2017). Recently, innovative technologies, including web-based, mobile and web applications, have increasingly been used as a medium for gathering and exchanging health information. Wolpin et al., (2010) developed an open-source web application that measures user reaction times, subjective quality ratings, and accuracy in completing tasks across different audio files created by text-to-speech engines. This study was successful in building and piloting the web application for a health intervention to improve users’ knowledge and health practices. In addition, mobile and web applications have been used to increase health screening tests for *O. viverrini *infection. The study showed that compared to laboratory tests, these tools are simple, rapid and low cost (Kaewpitoon et al., 2018). Internet-based health technology, including web-based resources, patient portals, and mobile phone apps, is increasingly being used to help manage chronic disease, educate people, and monitor users’ condition (Madrigal and Escoffery, 2019). The participants have been highly satisfied and use such technology frequently. Khademian et al (2020) assessed web-based health information seeking and eHealth literacy among Iranian college students and showed that participants usually searched for illnesses, symptoms, and treatments after they became sick and paid little attention to other aspects related to integral health. Prachaiboon et al (2021) conducted a cross-sectional analytical study of HL among 1,163 respondents from Northeast Thailand and found that they had inadequate access to health information, inadequate understanding of health information, inadequate appraisal of health information and inadequate health information associated with raw cyprinoid fish consumption. This study indicated an urgent need to improve *O. viverrini *information, particularly in HL, and change people’s eating behaviors, traditions, and contexts, including feeding dogs and cats. Therefore, the effects of health literacy promotion programs and OVCCA web applications on *O. viverrini *prevention were examined in this study. The intervention group was assigned to receive information on *O. viverrini *through HL and an OVCCA web application, while the control group received general information on *O. viverrini *from local health personnel as an annual campaign. The results reveal that the intervention can increased the participants’ knowledge, ability to access information, ability to understand information, ability to appraise information, ability to apply information and motivation for protection from *O. viverrini *infection. These results were similar to those of previous studies about other diseases; in particular, Puttapunyo et al., (2016) reported the effects of health behavioral development programs on the HL and weight-loss behavior of overweight personnel at Roi Et Hospital, Mueang District, Roi Et Province, Thailand. The study revealed that after the experiment, the participants had significantly higher HL and weight loss behavior than before the experiment, and the difference was statistically significant. Furthermore, Padchasuwan et al., (2018) conducted a study of secondary school students in Northeast Thailand and suggested that HL can be a desirable strategy for informing those with a lower practice level regarding liver fluke prevention and control. Continuous and extensive health education programs help people obtain more information on diseases and increase their HL.

Our study found that the participants in the intervention group had increased HL regarding *O. viverrini *prevention and control after they received information on *O. viverrini *during a 6-week health behavioral development program with 15 activities through the HL program and OVCCA web application. This result is similar to that of Boom (1971), who suggested that a successful behavioral modification program should be composed of a variety of activities. Our program included lectures using PowerPoint, posters, brochures, pamphlets, liver fluke slides under a microscope, VDO clips, daily health behavior records, idea sharing, safety cooking demonstrations, and local folk songs. The program was advantageous in helping the participants understand more information and motivating them to prevent *O. viverrini *infection. In addition, the activities, including discussion of *O. viverrini *information and idea sharing by role models who had succeeded in liver fluke prevention and control, supported behavioral change and HL in this group. These results were similar to those of other studies on motivation for behavioral change. Iftikhal (2018) applied protection motivation theory in terms of disease prevention behaviors for liver fluke infection in Ban Nong Hoi grade school, Muang District, Sakon Nakhon Province, Thailand, by using variations on 4 activities. The study found that the group that received the program had higher mean scores for knowledge, perceived severity, perceived susceptibility, response efficacy, efficacy expectation, health promotion, and disease prevention behaviors for liver fluke than the control group at the level of significance. Panithanang et al., (2018) indicated that the experimental group that received a health behavioral modification program based on health education, self-efficacy, motivation, social support and networking had significantly higher scores than those in the comparison group. Participants gained the correct knowledge and had higher self-efficacy, expectations, and practices regarding liver fluke prevention. The educational intervention consisted of seven training sessions (introduction to cholangiocarcinoma, risk factors, complications, benefits and barriers to proper consumption of cooked fish, carcinogenic agents, behavioral protection, and self-efficacy in applying preventive behaviors), and the participants showed a significant increase in knowledge, perceived susceptibility, perceived severity, perceived benefits, perceived barriers, cues to action, self-efficacy, and cholangiocarcinoma preventive behaviors (Srithongklang et al., 2019). In our study, the intervention group received motivation and continued support for obtaining information and *O. viverrini *prevention through the OVCCA web application.

The HL program and OVCCA web application were advantageous in enabling the participants to access information and manage their health. The experimental group had better *O. viverrini *infection prevention practices than the control group. In the OVCCA web application, the contents comprised images, animations, clips, screening tests, and local folk songs that benefited users by improving their knowledge and management of their health. The varieties of content and materials that have proven to be advantageous have increasingly been technology based, mainly animations, clips and songs. An interactive animation program for improving the knowledge of students studying liver fluke showed that the students achieved a significantly improved perception. The use of the animated program facilitated education about liver fluke and was good supplementary learning material for the students, particularly when they were learning about serious concepts (Bukkhunthod et al., 2020). The success of our web application is similar to that of Wang et al., (2015), who successfully designed a recommendation-based mobile web application to assist patients in efficiently seeking online health information at any time, anywhere and via any devices using a collaborative filtering approach to recommend health information. Carrasco-Hernandez et al., (2020) reported the long-term efficacy of a mobile app supporting psychopharmacological therapy for smoking cessation. They also performed a complementary assessment of the applied innovative technology and found that the proposed mobile health solution complementing psychopharmacological therapy showed greater efficacy in achieving 1-year tobacco abstinence than psychopharmacological therapy alone. This result suggests that such technology provides a basis for future artificial intelligence-based approaches. In addition, Ferrucci et al., (2021) reported that a specific web-based mobile app provided adequate and personalized support to complex health care populations. The results showed good family and community engagement and revealed some limitations that need to be addressed. Patients described themselves as satisfied or very satisfied. Health care providers reported improved communication with colleagues and the need to support data quality. With increased access to the internet and technology, there are many opportunities for utilizing the internet, electronic health applications, and information for the prevention and management of diseases. Our results show that an OVCCA web application is beneficial for promoting health and disease prevention, particularly HL in terms of *O. viverrini*. 

In conclusion, this study showed that the health literacy promotion program and OVCCA web application for the prevention of *O. viverrini* infection could serve as a potential strategy and guideline for self-care and health promotion for the prevention of *O. viverrini *infection in Northeast Thailand. In addition, it indicated that the program and OVCCA web application could enable users to access information, understand information, make decisions and appraise information, apply information and practice prevention of *O. viverrini* infection.

## Author Contribution Statement

Study conception and design: OW, TP, NKR, PB; data collection: TP, TM, SB; analysis and interpretation of results: OW, TP, NKR, SWR, PB, TK; draft manuscript preparation: OW, TP, NKR, SWR, PB; All authors reviewed the results and approved the final version of the manuscript. 
